# A 3D-2D Multibranch Feature Fusion and Dense Attention Network for Hyperspectral Image Classification

**DOI:** 10.3390/mi12101271

**Published:** 2021-10-18

**Authors:** Hongmin Gao, Yiyan Zhang, Yunfei Zhang, Zhonghao Chen, Chenming Li, Hui Zhou

**Affiliations:** 1 Key Laboratory of Water Big Data Technology of Ministry of Water Resources, Hohai University, No. 8 Focheng Road, Nanjing 211100, China; gaohongmin@hhu.edu.cn (H.G.); zhangyiyan@hhu.edu.cn (Y.Z.); yunfeizhang@hhu.edu.cn (Y.Z.); chenzhonghao@hhu.edu.cn (Z.C.); lcm@hhu.edu.cn (C.L.); 2 College of Computer and Information, Hohai University, No. 8 Focheng Road, Nanjing 211100, China; 3 College of Computer and Software, Nanjing Vocational University of Industry Technology, Nanjing 211100, China

**Keywords:** convolutional neural network, hyperspectral image classifications, multibranch feature fusion, dense attention block

## Abstract

In recent years, hyperspectral image classification (HSI) has attracted considerable attention. Various methods based on convolution neural networks have achieved outstanding classification results. However, most of them exited the defects of underutilization of spectral-spatial features, redundant information, and convergence difficulty. To address these problems, a novel 3D-2D multibranch feature fusion and dense attention network are proposed for HSI classification. Specifically, the 3D multibranch feature fusion module integrates multiple receptive fields in spatial and spectral dimensions to obtain shallow features. Then, a 2D densely connected attention module consists of densely connected layers and spatial-channel attention block. The former is used to alleviate the gradient vanishing and enhance the feature reuse during the training process. The latter emphasizes meaningful features and suppresses the interfering information along the two principal dimensions: channel and spatial axes. The experimental results on four benchmark hyperspectral images datasets demonstrate that the model can effectively improve the classification performance with great robustness.

## 1. Introduction

With the development of remote sensing, hyperspectral imaging technology has been widely applied in meteorological warning [1], agricultural monitoring [2], and marine safety [3]. Hyperspectral images are composed of hundreds of spectral bands and contain rich land-cover information. Hyperspectral image classification has received increasing attention as a crucial issue in the field of remote sensing.

The conventional classification methods include random forest (RF) [4], multiple logistic regression (MLP) [5], and support vector machine (SVM) [6]. They are all classified based on one-dimensional spectral information. Additionally, principal component analysis (PCA) [7] tends to be used to compress spectral dimensions while retaining essential spectral features, reduce band redundancy, and improve model robustness. Although these traditional methods obtain great results, they have a limited representation capacity and can only extract low-level features due to the shallow nonlinear structure.

Recently, hyperspectral images classification methods based on deep learning (DL) [8,9,10] have been increasingly favored by researchers to make up for the shortcomings of traditional methods. CNN has made a great breakthrough in the field of computer vision due to its excellent image representation ability and has been proved successful in the field of hyperspectral image classification. Makantasis et al. [11] developed a network based on 2D CNNs, where each pixel was packed into image patches of fixed size for spatial feature extraction and sent to multilayer perceptron for classification. However, 2D convolution can only extract features in height and width dimensions, ignoring the rich information of spectral bands. To further enhance the utilization of the spectral dimensions, the researchers turned their attention to 3D CNNs [12,13,14]. He et al. [12] proposed a multiscale 3D deep convolutional neural network (M3D-DCNN) for HSI classification, which learns the spatial and spectral features in the raw data of hyperspectral images in an end-to-end manner. Zhong et al. [13] developed a 3D spectral-spatial residual network (SSRN) that continuously learns discriminative features and spatial context information from redundant spectral signatures. Although 3D CNNs can make up for the defects of 2D CNNs in this regard, while 3D CNNs introduce a large number of computational parameters, increasing the training time and memory cost. In addition, due to the HSI having the characteristics of solid correlations between bands, there are phenomena of the same material that may present spectral dissimilarity. Different materials may have homologous spectral features, which seriously interfere with the extraction of spectral information and lead to the degradation of classification performance. How to distinguish the discriminative features of HSI is the key to improving the classification performance. Recent studies have shown that discriminative features can be enhanced using attention mechanisms [15,16]. Studies in cognitive biology reveal that human beings acquire the information of significance by paying attention to only a few critical features while ignoring the others. Similarly, attention has been effectively applied to various tasks in computer vision [17,18]. Numerous approaches based on existing attention mechanisms are also involved in the hyperspectral image classification tasks, demonstrating their efficiency in improving performance.

Additionally, with the continuous deepening of feature extraction, the neural network will inevitably become deeper. The phenomenon of gradient vanishing and network degradation becomes more and more serious, which deteriorates the classification performance. Furthermore, screening the crucial features from the complex features of hyperspectral images has become critical for network performance improvement. The paper proposed a 3D-2D multiscale feature fusion and dense attention network (MFFDAN) for hyperspectral image classification considering the above problems. In summary, the main contributions of this paper are as follows:

(1) The whole network structure is composed of 3D and 2D convolutional layers, which not only avoids the problems of insufficient feature extraction when only 2D CNNs are used but also reduces the large number of training parameters caused by 3D CNNs alone, thereby improving the model efficiency.
(2) The proposed 3D multiscale feature fusion module obtains the scaled features by combining multiple convolutional filters. In general, filters with large sizes are unable to capture the fine-grained structure of the images, whereas filters with small sizes are frequently eliminate the coarse-grained features of the images. Combining multiple convolutional filters of varying sizes allows for the extraction of more detailed features.
(3) A 2D densely connected attention module is developed to overcome the gradient vanishing problem and select discriminative channel-spatial features from redundant hyperspectral images. A factorized spatial-channel attention block is proposed that can adaptively prioritize critical features and suppress less useful ones. Additionally, a simple 2D dense block is introduced to facilitate the information propagation and feature reuse and comprehensively utilizes features of different scales in 3D HSI cubes.

The rest of this paper is arranged as follows: Section 2 introduces the related CNNs methods and the frameworks of MFFDAN. Section 3 shows specific experiments on four benchmark datasets. Finally, the conclusions are presented in Section 4.

## 2. Material and Methods

### 2.1. D Multibranch Fusion Module

Three-dimensional CNNs work on hyperspectral images’ spectrum and spatial dimensions simultaneously through 3D convolution kernels and can directly extract spatial and spectral information from the raw hyperspectral images. The formula is as follows:

(1) $$V_{l,i}^{x,y,z}=f(\sum_{m}\sum_{h=0}^{H_{l}-1}\sum_{w=0}^{W_{l}-1}\sum_{r=0}^{D_{l}-1}k_{l,i,m}^{h,w,d}v_{l-1,m}^{x+h,y+w,z+d}+b_{l,i})$$

where $H_{l}$, $W_{l}$, and $D_{l}$ represents the height, width, and spectral dimension of convolution kernels. The $k_{l,i,m}^{h,w,d}$ denotes the output value of the i-th convolution kernel in the l-th layer at the position of (h,w,d).

The normal 3D CNN methods for hyperspectral image classification involve stacking convolutional blocks of convolutional layers (Conv), batch normalization (BN), and activation functions to extract detailed and discriminative features from raw hyperspectral images. While these methods improve the classification results to a certain degree, they also introduce numerous calculating parameters and increase the training time. Additionally, building deep convolutional neural networks tends to cause gradients vanishing and to suffer from classification performance degradation.

**Figure 1:**
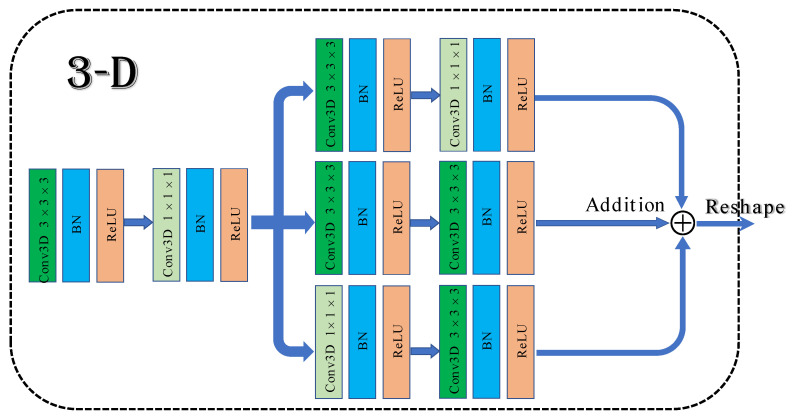
The architecture of 3D multibranch fusion module.

To solve the above problems, a 3D multibranch fusion module is proposed in this work. The architecture of the module is shown in Figure 1. First, 3 × 3 × 3 and 1 × 1 × 1 convolutional blocks are employed to form the shallow network, which can expand the information flow and allow the network to learn texture features. Then, it adds three branches that are composed of multiple convolution kernels in sequence. Different sizes of convolutional filers can be used to extract multiscale features from hyperspectral data. Merging with the shallow network frequently results in superior classification performance compared to stacked convolutional layers.

### 2.2. D-2D CNN

On the one hand, the features extracted by 2D CNNs alone are limited. On the other hand, 3D CNN consumes a substantial amount of computational resources. The combination of 2D CNNs and 3D CNNs can effectively make up for these defects. Roy et al. [14] proposed a hybrid spectral-spatial neural network HybridSN. First, 3D CNNs facilitate the joint spatial-spectral feature representation from a stack of spectral bands. Then, the 2D CNNs on top of the 3D CNNs further learn more abstract-level spatial representation. Compared with 3D CNNs alone, the hybrid CNNs can not only avoid the problem of insufficient feature extraction but also reduce model training parameters and improve model efficiency.

### 2.3. Attention Mechanism

#### 2.3.1. Channel Attention

**Figure 2:**
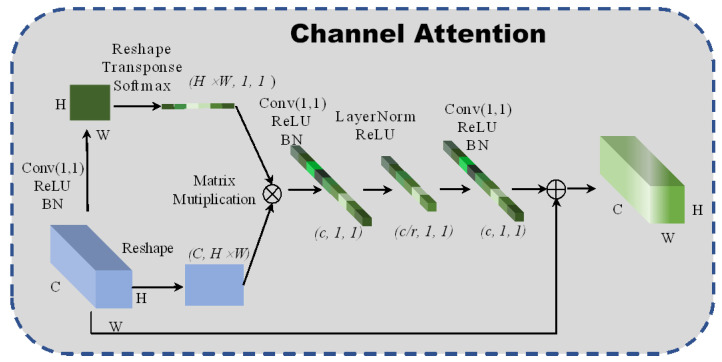
The architecture of 2D channel attention block.

A new global context channel attention block is designed to enable the network to pay attention to the relationship between adjacent pixels in the channel dimensions. Simultaneously, the channel attention block also gains a long-distance dependence between pixels and improves the network’s global perception. The structure of the spectral attention is shown in Figure 2. The fully connected layer increases computational parameters. For this reason, a 1 × 1 convolutional neural network is intended to replace the fully connected layers, in which the scaling factor is set to 4 to reduce the calculation cost and prevent the network from overfitting. Additionally, layer normalization [19] is introduced to sparse the weights of the network:

(2) $${\hat{z}}^{\left(l\right)}=\frac{z^{\left(l\right)}-\mu^{\left(l\right)}}{\sqrt{\sigma^{{\left(l\right)}^{2}}+\varepsilon }}⊙\gamma +\beta ≜LN_{\gamma ,\beta }(z^{\left(l\right)})$$

where γ and β represent the parameter vectors of zooming and translation, respectively. The layer normalization is used to normalize the weights’ matrix, which can accelerate the convergence and regularization of the network. The formula of the channel attention is:

(3) $$Z_{i}=X_{i}+W_{v2}ReLU(LN(W_{v1}\sum_{j=1}^{N_{P}}\frac{e^{W_{k}x_{j}}}{\sum_{m=1}^{N_{p}}e^{W_{k}x_{m}}}x_{j}))$$

where $\partial_{j}=\frac{e^{W_{k}x_{j}}}{\sum_{m}e^{W_{k}x_{m}}}$ represents the global pooling and $W_{v2}ReLU(LN(W_{v1}(\cdot )))$ denotes the bottleneck transform. The channel attention module uses global attention pooling to model the long-distance dependences and capture discriminative channel features from the redundant hyperspectral images.

#### 2.3.2. Spatial Attention

**Figure 3:**
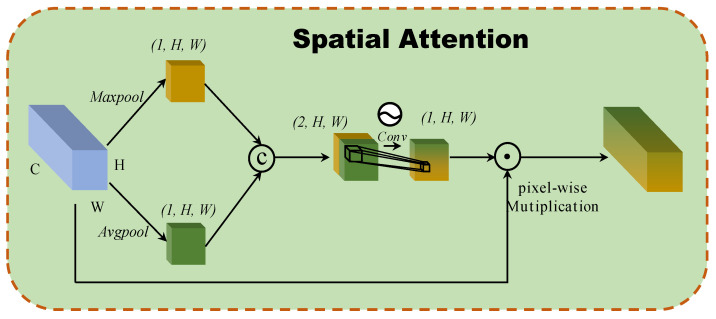
The architecture of 2D spatial attention block.

A spatial attention block based on the interspatial relationships of features is developed, as inspired by CBAM [20]. Figure 3 illustrates the structure of the spatial attention block. To generate an efficient feature descriptor, average-pooling and max-pooling operations are applied along the channel axis, and they concatenate them. Pooling operations along the channel axis are shown to be effective at highlighting informative regions. Then, a convolution layer is applied to the concatenated feature descriptor to create a spatial attention map that specifies which features to emphasize or suppress. These are then convolved using a standard convolution layer to create a two-dimensional spatial attention map. In short, spatial attention is calculated as follows:

(4) $$\begin{array}{ll} M(F) & =\sigma (f^{3\times 3}[AvgPool(F);MaxPool(F)])\\ & =\sigma (f^{3\times 3}([F_{avg};F_{\max}])) \end{array}$$

where σ denoted the sigmoid function and $f^{3\times 3}$ represents a convolution operation with the filter size of 3×3.

### 2.4. HSI Classification Based on MFFDAN

**Figure 4:**
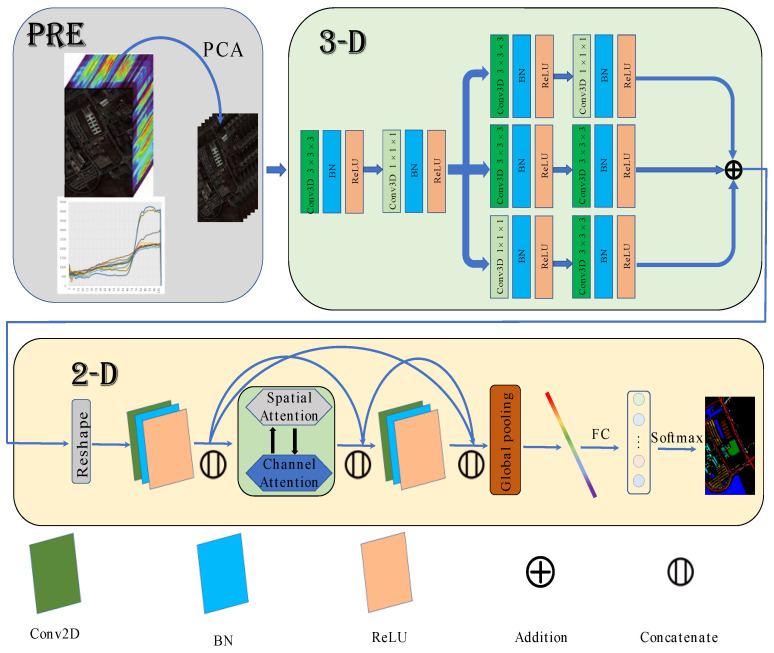
The Flowchart of MFFDAN network.

The architecture of MFFDAN is depicted in Figure 4. The University of Pavia dataset is used to demonstrate the algorithm’s detailed process. The raw data are normalized to zero mean value with unit variance for preprocessing. Then, the PCA is employed to compress the spectral dimensions and eliminate band noise in the raw HSIs. Finally, hyperspectral image data were segmented into the fixed spatial size of 3D image patches centered on labeled pixels. After completing the above steps, they are sent to the 3D multibranch fusion module for feature extraction. The module is intended to extract multiscale features with multiple sizes of convolutional filters. Following that, the 3D feature maps reshape to 2D after dimension transformation and are sent to the 2D dense attention module. Then, a dense block [21] is arranged with spatial and channel attention in the middle of the module, which is used to enhance the information flow and adaptively select out the discriminative spatial-channel features. Finally, a fully connected layer with a softmax function is used for classification.

## 3. Results

### 3.1. Datasets

The University of Pavia (PU) dataset was acquired by the reflective optics system imaging spectrometer (ROSIS) sensor. The dataset consists of 103 spectral bands. There are 610 × 340 pixels and nine ground-truth classes in total. The number of training, validation, and testing samples in experiments is given in Table 1.

The Kennedy Space Center (KSC) dataset was gathered in 1996 by AVIRIS with wavelengths ranging from 400 to 2500 nm. The images have a spatial dimension of 512 × 614 pixels and 176 spectral bands. The KSC dataset consists of in total 5202 samples of 13 upland and wetland classes. The number of training, validation, and test samples in experiments is given in Table 2.

The Salinas Valley (SA) dataset contains 512 × 217 pixels with a spatial resolution of 3.7 m and 224 bands with wavelengths ranging from 0.36 to 2.5 μm. Additionally, 20 spectral bands of the dataset were eliminated due to water absorption. The SA dataset contains 16 labeled material classes in total. The number of training, validation, and testing samples in experiments is given in Table 3.

The Grass_dfc_2013 dataset was acquired by the compact airborne spectrographic imager (CASI) over the campus of the University of Houston and the neighboring urban area [22]. The dataset contains 349 × 1905 pixels, with a spatial resolution of 2.5 m and 144 spectral bands ranging from 0.38 to 1.05 µm. It includes 15 classes in total. The number of training, validation, and testing samples in experiments is given in Table 4.

### 3.2. Experimental Setup

The model proposed in this paper is implemented base on Python language and Pytorch deep learning framework. All experiments are carried out on a computer with Windows 10 operating system, NVIDIA RTX 2060 Super GPU, and 64 GB RAM. The overall accuracy (OA), average accuracy (AA), and kappa coefficient (Kappa) are adopted as the evaluation criteria. Different proportions of training, validation, and testing samples for each dataset are used to verify the effectiveness of the proposed model considering the unbalanced categories in four benchmarks.

The batch size and epochs are set to 16 and 200, respectively. Stochastic gradient descent (SGD) is adopted to optimize the training parameters. The initial learning rate is 0.05 and decreases by 1% every 50 epochs. All the experiments are repeated five times to avoid errors.

### 3.3. Analysis of Parameters

**Figure 5:**
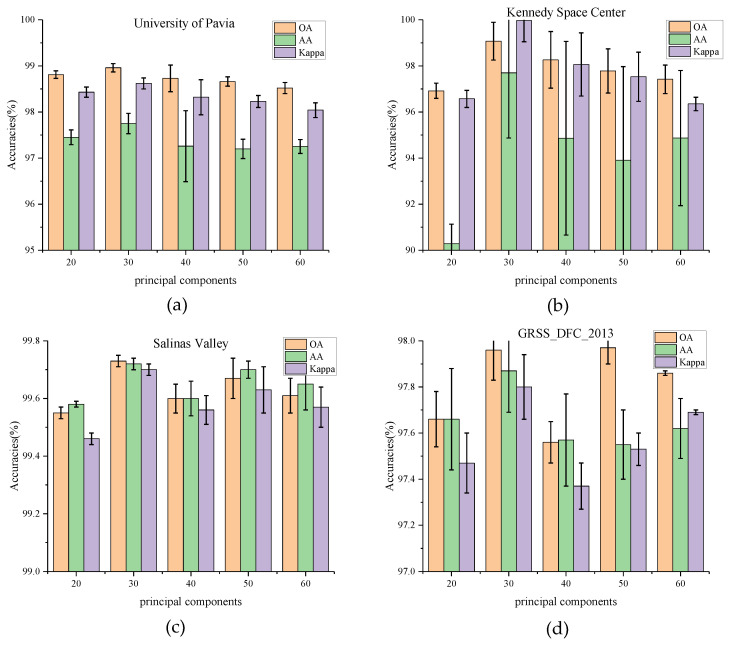
OA, AA, and Kappa accuracies with different principal components on four datasets. (**a**) Effect of principal components on University of Pavia dataset, (**b**) Effect of principal components on Kennedy Space Center, (**c**) Effect of principal components on Salinas Valley dataset, (**d**) Effect of principal components on GRSS_DFC_2013 dataset.

(1) Impact of Principal Component: In this section, the influence of the number of principal components C is tested on classification results. PCA is first used to reduce the dimensionality of the bands to 20, 30, 40, 50, and 60, respectively. The experimental results on four datasets are shown in Figure 5. For the University of Pavia and Kennedy Space Center datasets, the values of OA, AA, and Kappa rise from 20 (PU_OA = 98.81%, KSC_OA = 96.92%) and reach a peak at 30(PU_OA = 98.96%, KSC_OA = 99.07%). The increase in OA values on the KSC dataset is much higher than that on the PU dataset. It can be observed that the number of principal components has a significant impact on the KSC dataset. When the principal component bands exceed 30, these indicators decline to vary degrees. While for the Salinas Valley and GRSS_DFC_2013, the values of OA, AA, and Kappa seem to have no such relationships with the principal components. The OA values fluctuate in various number of principal components. The phenomenon is most likely caused by the fact that the latter two datasets have a higher land-cover resolution but a lower spectral band sensitivity.

(2) Impact of Spatial Size: The choice of the spatial size of the input image block has a crucial influence on classification accuracy. To find the best spatial size, it is necessary to test the model by adopting different spatial sizes: C × 9 × 9, C × 11 × 11, C × 13 × 13, C × 15 × 15, C × 17 × 17, and C × 19 × 19, where C is the fixed number of the principal component. The number of the principal component is set to 30 in all experiments to guarantee fairness.

**Figure 6:**
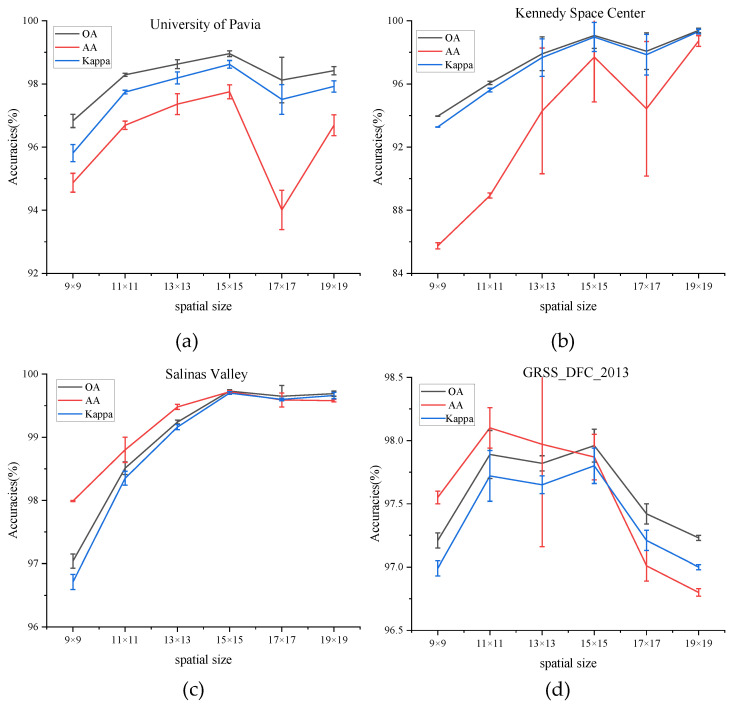
OA, AA, and Kappa accuracies with different spatial size on four datasets. (**a**) Effect of spatial size on University of Pavia dataset, (**b**) Effect of spatial size on Kennedy Space Center dataset, (**c**) Effect of spatial size on Salinas Valley dataset, (**d**) Effect of spatial size on GRSS_DFC_2013 dataset.

Figure 6 shows the values of OA, AA, and Kappa of different spatial sizes on four datasets. The values of OA, AA, and Kappa rise steadily from spatial sizes of C × 9 × 9 to C × 15 × 15 on PU, KSC, and SA dataset and then decrease in the larger spatial sizes. That is to say, a target pixel and its adjacent neighbors usually belong to the same class to certain spatial sizes, while oversized regions may present additional noise and deteriorate the classification performance. While for the dataset of GRSS_DFC_2013, the values of three indicators fluctuate between the spatial size of C × 11 × 11 and C × 15 × 15 and decrease with larger spatial sizes. To sum up, the size of the patch for all datasets is set to C × 15 × 15.

### 3.4. Ablation Study

**Figure 7:**
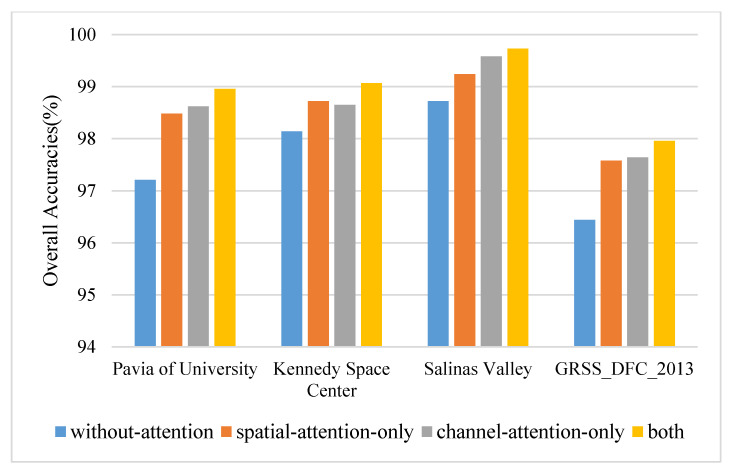
The overall accuracies of ablation experiments on four datasets.

In order to test the effectiveness of the proposed densely connected attention module, several specific ablation experiments are designed. The models used for comparison are consistent with the network of the proposed method except for the removal of the densely connected attention module. The principal components and the spatial size are set to 30 and 15 × 15. The results on four datasets are displayed in Figure 7. The densely connected attention module improves the values of OA by approximately 0.93–1.75% on four datasets. Specifically, on most occasions (such as PU, SA, and GRSS dataset), a single-channel attention block outperforms a single spatial attention block by approximately 0.06–0.34% OA values. However, that does not mean that the spatial attention mechanism does not work, which plays a significant role in improving classification performance. Spatial attention alone has improved (0.52–1.27% OA) compared with nonattention block. The reason is likely that the densely connected attention module introduces the combination of attention mechanisms and densely connected layers. On the one hand, the attention mechanism can adaptively assign different weights to spatial-channel regions and suppress the effects of interfering pixels. On the other hand, densely connected layers relieve the gradient vanishing when the model bursts into deep layers and enhances the feature reuse during the convergence of the network.

### 3.5. Compared with Other Different Methods

To evaluate the performance of the proposed method, seven classification methods were selected to be compared. The methods are RBF-SVM with radial basis function kernel, multinomial logistic regression (MLR), random forest, spatial CNN with 2-D kernels [11], PyResNet [23], Hybrid-SN [14], and SSRN [13]. Figure 5, Figure 6 and Figure 7 show the classification maps of different methods on PU, KSC, SA, and Grss_dfc_2013 datasets.

The spatial size and the number of principal components are set to C × 15 × 15 and 30 for all DL methods to guarantee fairness. All the comparison experiments are carried out five times and calculate the average values and standard deviations. Due to the SSRN model not performing PCA in the manner described in the original paper, the results are the same when omitting this process in experiments. Other hyperparameters of the network are configured according to their papers.

**Figure 8:**
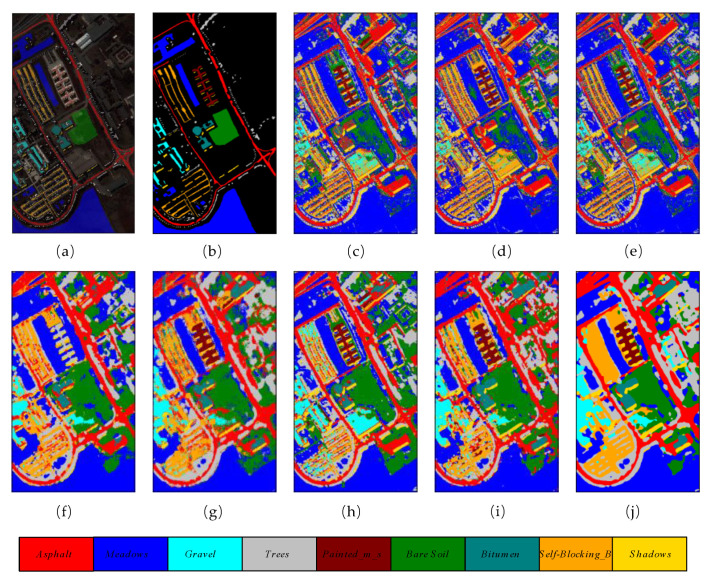
The classification maps of different methods on University of Pavia dataset. (**a**) False color map with truth labels, (**b**) ground truth, (**c**) RBF-SVM (**d**), MLR (**e**), RF (**f**) 2D-CNN (**g**), PyResNet (**h**) SSRN, (**i**) HybridSN, and (**j**) proposed.

The number of training, validation, and testing samples on University of Pavia dataset for comparison are in accordance with the list of samples in Table 1. Table 5 reveals the overall accuracy, average accuracy, and kappa coefficient of the different methods. It is obvious that classic machine learning methods such as RBF-SVM, RF, and MLR achieve relatively lower overall accuracies compared with other DL methods. They classify through the spectral dimensions of HSIs, which ignore the importance of 2D spatial characteristics. The proposed method obtained the best results among all the comparison methods, with 98.96% overall accuracy, which is 1.63% higher than the second-best (97.33%) achieved by HybridSN. Figure 8 shows the classification maps of these methods.

The selection of samples for training, validation, and testing on Kennedy Space Center dataset are consistent with the list of samples in Table 2. It is necessary to increase the training samples for the KSC dataset to avoid the underfit of the network. The 2D CNN model achieves the worst results among all the DL methods, which is difficult to obtain complex spectral-spatial features via 2D convolutional filters. The SSRN model obtains the second-best results due to its stacked 3D convolutional layers, which extract the discriminative spectral-spatial features from raw images.

**Figure 9:**
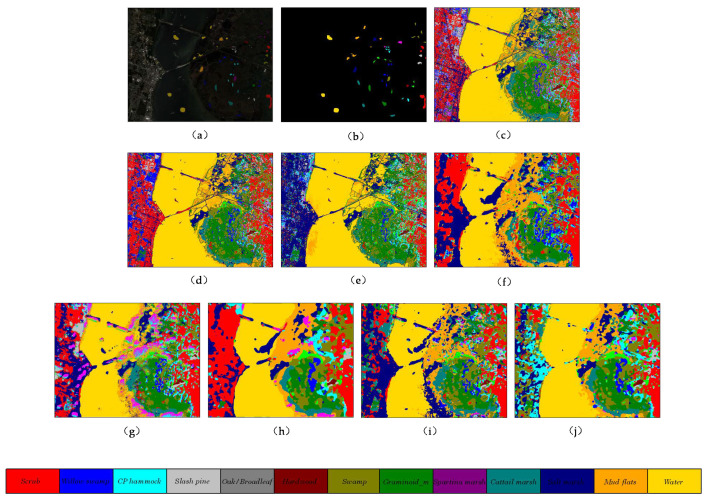
The classification maps of different methods on Kennedy Space Center dataset. (**a**) false color map with truth labels, (**b**) ground truth, (**c**) RBF-SVM, (**d**) MLR, (**e**) RF, (**f**) 2D-CNN, (**g**) PyResNet, (**h**) SSRN, (**i**) HybridSN, and (**j**) proposed.

The proposed method achieves the best results with (OA = 99.07%, AA = 97.70%, and Kappa = 98.97%). The quantitative classification results in terms of three indices, and the accuracies for each class are reported in Table 6. The classification maps of these methods are displayed in Figure 9.

**Figure 10:**
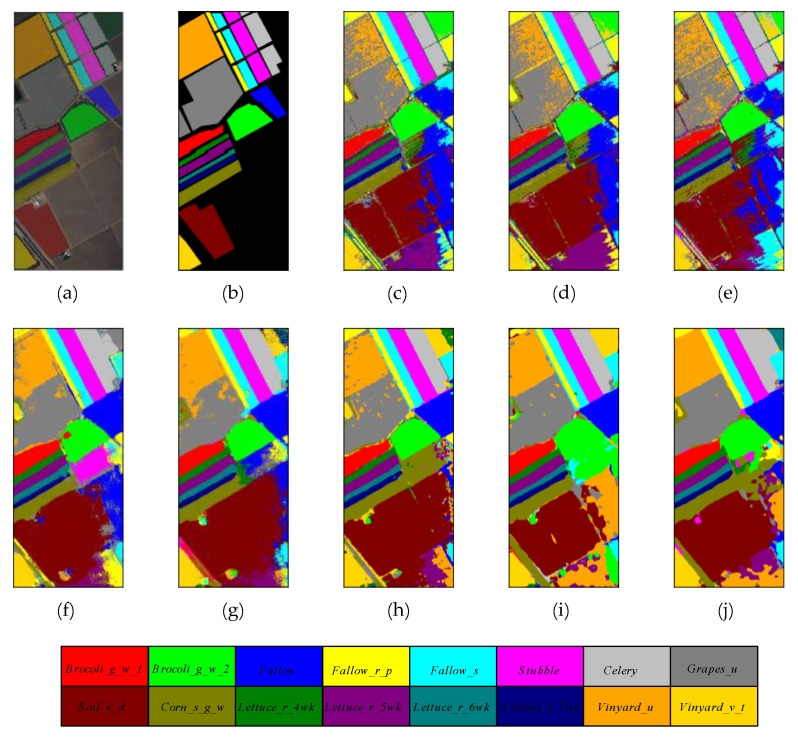
The classification maps of different methods on Salinas Valley dataset. (**a**) False color map with truth labels, (**b**) ground truth, (**c**) RBF-SVM, (**d**) MLR, (**e**) RF, (**f**) 2D-CNN, (**g**) PyResNet, (**h**) SSRN, (**i**) HybridSN, and (**j**) proposed.

**Figure 11:**
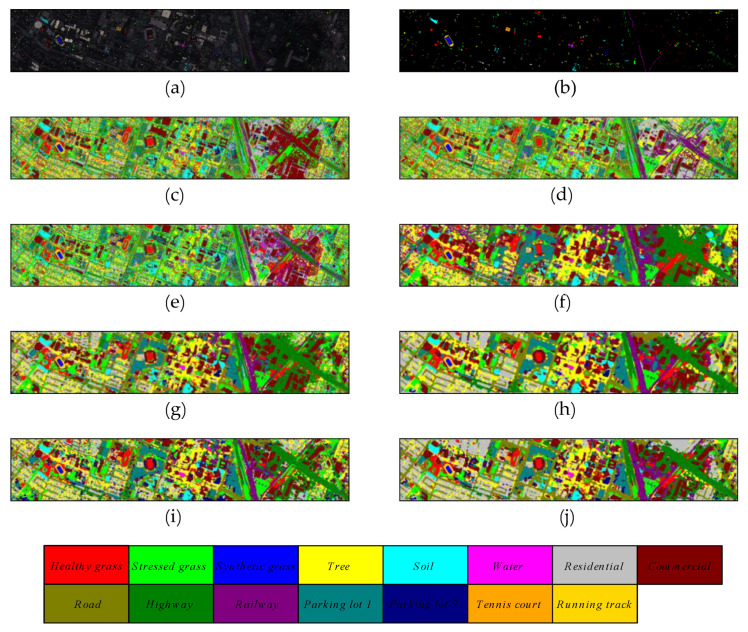
The classification maps of different methods on Grass_dfc_2013 dataset. (**a**) false color map with truth labels, (**b**) ground truth, (**c**) RBF-SVM, (**d**) MLR, (**e**) RF, (**f**) 2D-CNN, (**g**) PyResNet, (**h**) SSRN, (**i**) HybridSN, and (**j**) proposed.

The selection of samples for training, validation, and testing on Salinas Valley and Grass_dfc_2013 datasets is consistent with the list of samples in Table 3 and Table 4. Meanwhile, the quantitative results of different methods on these two datasets are reported in Table 7 and Table 8, respectively. The proposed method outperforms other comparison methods in terms of OA, AA, and Kappa indicators. The 3D multibranch feature fusion module can extract the multiscale features from raw hyperspectral images and improve the performance significantly. Figure 10 and Figure 11 reveal the classification maps of methods on these two datasets, which clearly show that the proposed model has better visual impressions than other comparison methods. For other models, the HybridSN and SSRN models have better classification performance than traditional machine learning methods and shallow DL classifiers. Specifically, the HybridSN model achieves 98.97% in OA, 98.95% in terms of AA, and 98.85% in terms of Kappa on the SA dataset, demonstrating the excellent feature representation ability in deep neural networks. SSRN model achieves 97.62% in OA, 98.47% in AA, and 97.36% in Kappa. The large kernel filters are good at extracting original features from HSIs without the PCA process. By comparison, the shallow 2D classifiers such as 2D CNN and PyResNet cannot obtain comprehensive features and miss rich spectral information during the training process. Therefore, they do not achieve competitive classification performance as HybridSN and SSRN models.

## 4. Conclusions

In this paper, a novel deep learning method called 3D-2D multibranch feature fusion and dense attention network is proposed for hyperspectral images classification. Both 3D and 2D CNNs are combined in an end-to-end network. Specifically, the 3D multibranch feature fusion module is designed to extract multiscale features from the spatial and spectrum of the hyperspectral images. Following that, a 2D dense attention module is introduced. The module consists of a densely connected block and a spatial-channel attention block. The dense block is intended to alleviate gradient vanishing in deep layers and enhance the reuse of features. The attention module includes the spatial attention block and the spectral attention block. The two blocks can adaptively select the discriminative features from the space and the spectrum of redundant hyperspectral images. Combining the densely connected block and attention block can significantly improve the classification performance and accelerate the convergence of the network. The elaborate hybrid module raises the OA by 0.93–1.75% on four different datasets. Additionally, the proposed model outperforms other comparison methods in terms of OA by 1.63–18.11% on the PU dataset, 0.26–16.06% on the KSC dataset, 0.76–13.48% on the SA dataset, and 0.46–23.39% on the Grass_dfc_2013 dataset. These experimental results demonstrate that the model proposed can achieve satisfactory classification performance.

## Figures and Tables

**Table 1:** The number of training, validation and testing samples for University of Pavia dataset.

No.	Name	Train	Val	Test
1	Asphalt	66	657	5908
2	Meadows	186	1846	16,617
3	Gravel	21	208	1870
4	Trees	31	303	2730
5	Painted-m-s	13	133	1199
6	Bare Soil	50	498	4481
7	Bitumen	13	132	1185
8	Self-B-Bricks	37	365	3280
9	Shadows	9	94	844
	Total	426	4236	38,114

**Table 2:** The number of training, validation, and testing samples for Kennedy Space Center dataset.

No.	Name	Train	Val	Test
1	Scrub	76	69	616
2	Willow swamp	24	22	197
3	CP hammock	26	23	207
4	Slash pine	25	23	204
5	Oak/Broadleaf	16	15	130
6	Hardwood	23	21	185
7	Swamp	11	9	85
8	Graminoid marsh	43	39	349
9	Spartina marsh	52	47	421
10	Cattail marsh	40	36	328
11	Salt marsh	42	38	339
12	Mud flats	50	45	408
13	Water	93	83	751
	Total	521	470	4220

**Table 3:** The number of training, validation, and testing samples for Salinas Valley dataset.

No.	Name	Train	Val	Test
1	Brocoli_green _1	20	199	1790
2	Brocoli_green _1	37	369	3320
3	Fallow	20	196	1760
4	Fallow _plow	14	138	1242
5	Fallow_smooth	27	265	2386
6	Stubble	40	392	3527
7	Celery	36	354	3189
8	Grapes_untrained	113	1116	10,042
9	Soil _develop	62	614	5527
10	Corn _weeds	33	325	2920
11	Lettuce _4wk	11	106	951
12	Lettuc _5wk	19	191	1717
13	Lettuce _6wk	9	91	816
14	Lettuce _7wk	11	106	953
15	Vinyard_untrain	73	720	6475
16	Vinyard _trellis	18	179	1610
	Total	543	5361	48,225

**Table 4:** The number of training, validation, and testing samples for Grass_Dfc_2013 dataset.

No.	Name	Train	Val	Test
1	Healthy grass	125	113	1013
2	Stressed grass	125	113	1016
3	Synthetic grass	70	63	564
4	Tree	124	112	1008
5	Soil	124	112	1006
6	Water	33	29	263
7	Residential	127	114	1027
8	Commercial	124	112	1008
9	Road	125	113	1014
10	Highway	123	110	994
11	Railway	124	111	1000
12	Parking lot 1	123	111	999
13	Parking lot 2	47	42	380
14	Tennis court	43	39	346
15	Running track	66	59	535
	Total	1503	1353	12,173

**Table 5:** The categorized results of different methods on the Paiva of University dataset.

Class	Methods							
	Conventional Classifiers			Classic Neural Networks				Proposed
	RBF-SVM	MLR	RF	2D-CNN	PyResNet	SSRN	HybridSN	
1	89.00 ± 1.10	90.21 ± 1.56	86.11 ± 2.21	93.30 ± 1.62	93.45 ± 1.14	99.19 ± 0.59	97.64 ± 1.37	**99.50 ± 0.09**
2	98.10 ± 0.65	96.35 ± 1.64	96.03 ± 1.23	99.39 ± 0.82	99.45 ± 0.50	98.18 ± 3.20	99.65 ± 0.22	**99.97 ± 0.02**
3	60.47 ± 5.17	42.36 ± 2.17	30.19 ± 3.89	71.09 ± 3.83	77.90 ± 2.74	85.28 ± 15.81	78.24 ± 4.87	**88.53 ± 0.90**
4	87.37 ± 4.35	79.68 ± 3.45	76.47 ± 5.17	94.30 ± 2.25	90.08 ± 2.95	95.85 ± 1.46	96.65 ± 0.32	**97.88 ± 0.30**
5	99.07 ± 0.32	98.89 ± 0.33	98.26 ± 0.44	33.26 ± 47.03	99.82 ± 0.09	**100.00 ± 0.00**	**100.00 ± 0.00**	**100.00 ± 0.00**
6	69.52 ± 3.50	50.48 ± 5.62	36.90 ± 4.85	95.24 ± 3.55	94.91 ± 1.41	96.42 ± 5.17	99.92 ± 0.10	**99.98 ± 0.03**
7	69.22 ± 12.02	5.82 ± 2.96	58.73 ± 6.89	57.18 ± 40.45	90.28 ± 0.75	94.85 ± 2.71	**98.19 ± 1.26**	97.92 ± 0.89
8	86.00 ± 3.04	87.85 ± 1.69	83.92 ± 5.04	87.33 ± 7.13	74.65 ± 8.45	84.49 ± 18.88	94.29 ± 2.79	**98.83 ± 0.23**
9	99.72 ± 0.08	99.47 ± 0.15	99.30 ± 0.20	19.30 ± 27.29	89.84 ± 1.86	99.57 ± 0.46	87.27 ± 9.37	**97.12 ± 1.04**
OA (%)	88.84 ± 0.34	82.77 ± 0.42	80.85 ± 0.69	90.00 ± 2.38	93.64 ± 0.62	96.14 ± 2.27	97.33 ± 0.45	**98.96 ± 0.09**
AA (%)	84.27 ± 1.65	72.34 ± 0.29	73.99 ± 1.40	72.26 ± 8.43	90.04 ± 0.71	94.87 ± 1.67	94.65 ± 1.04	**97.75 ± 0.22**
Kappa × 100	84.96 ± 0.50	76.50 ± 0.50	73.76 ± 0.89	86.53 ± 3.31	91.53 ± 0.82	94.90 ± 2.94	96.46 ± 0.60	**98.62 ± 0.12**

**Table 6:** The categorized results of different methods on the Kennedy Space Center dataset.

Class	Methods							
	Conventional Classifiers			Classic Neural Networks				Proposed
	RBF-SVM	MLR	RF	2D-CNN	PyResNet	SSRN	HybridSN	
1	95.85 ± 0.66	95.83 ± 0.79	94.63 ± 1.12	99.22 ± 1.00	99.42 ± 0.24	**100.00 ± 0.00**	99.48 ± 0.46	99.97 ± 0.06
2	85.39 ± 3.40	86.48 ± 2.06	81.19 ± 6.49	90.26 ± 4.42	88.43 ± 2.64	**100.00 ± 0.00**	95.52 ± 2.76	99.91 ± 0.18
3	88.09 ± 4.18	90.87 ± 4.98	89.48 ± 2.32	88.69 ± 3.39	96.52 ± 2.16	**100.00 ± 0.00**	92.26 ± 5.44	99.22 ± 0.80
4	42.47 ± 5.36	34.45 ± 6.04	68.11 ± 5.63	68.43 ± 1.62	65.20 ± 4.68	**95.30 ± 1.81**	81.14 ± 7.23	90.82 ± 5.06
5	47.45 ± 5.56	24.55 ± 11.17	46.48 ± 4.11	20.92 ± 29.59	66.44 ± 1.98	73.56 ± 3.10	77.38 ± 4.87	**98.01 ± 2.70**
6	47.67 ± 3.57	44.95 ± 2.56	37.09 ± 4.22	50.97 ± 36.05	90.13 ± 1.60	98.38 ± 1.65	95.83 ± 4.21	**99.61 ± 0.57**
7	83.58 ± 5.51	79.37 ± 7.81	75.79 ± 10.16	32.98 ± 46.65	**100.00 ± 0.00**	**100.00 ± 0.00**	97.05 ± 3.15	82.52 ± 34.95
8	90.77 ± 1.36	70.93 ± 4.96	72.58 ± 5.28	87.20 ± 2.37	99.40 ± 0.53	**100.00 ± 0.00**	**100.00 ± 0.00**	**100.00 ± 0.00**
9	94.53 ± 2.41	83.89 ± 1.50	92.78 ± 4.10	**100.00 ± 0.00**	99.86 ± 0.20	99.22 ± 1.11	99.91 ± 0.17	99.92 ± 0.10
10	92.69 ± 4.21	86.37 ± 30.35	81.81 ± 3.19	88.37 ± 2.79	97.25 ± 2.06	**100.00 ± 0.00**	**100.00 ± 0.00**	**100.00 ± 0.00**
11	96.98 ± 1.52	94.96 ± 1.43	95.81 ± 1.77	99.56 ± 0.63	98.67 ± 0.43	**100.00 ± 0.00**	99.73 ± 0.53	**100.00 ± 0.00**
12	85.83 ± 3.29	84.81 ± 2.61	82.21 ± 1.93	84.91 ± 9.16	70.71 ± 4.69	**100.00 ± 0.00**	98.23 ± 1.89	**100.00 ± 0.00**
13	99.93 ± 0.14	99.86 ± 0.18	99.74 ± 0.09	99.84 ± 0.23	**100.00 ± 0.00**	**100.00 ± 0.00**	**100.00 ± 0.00**	**100.00 ± 0.00**
OA (%)	87.59 ± 0.55	83.01 ± 0.69	84.87 ± 0.89	87.91 ± 1.77	92.84 ± 0.21	98.81 ± 0.17	97.28 ± 0.59	**99.07 ± 0.82**
AA (%)	80.86 ± 0.87	75.18 ± 0.86	78.28 ± 0.86	77.80 ± 5.35	90.16 ± 0.06	97.42 ± 0.19	95.12 ± 0.73	**97.70 ± 2.83**
Kappa×100	86.16 ± 0.61	81.03 ± 0.77	83.12 ± 0.98	86.51 ± 1.98	92.02 ± 0.24	98.67 ± 0.18	96.97 ± 0.65	**98.97 ± 0.92**

**Table 7:** The categorized results of different methods on the Salinas Valley dataset.

Class	Methods							
	Conventional Classifiers			Classic Neural Networks				Proposed
	RBF-SVM	MLR	RF	2D-CNN	PyResNet	SSRN	HybridSN	
1	97.28 ± 1.25	97.76 ± 0.69	97.07 ± 1.28	99.89 ± 0.15	99.77 ± 0.33	98.74 ± 0.93	99.99 ± 0.02	**100.00 ± 0.00**
2	99.53 ± 0.29	99.62 ± 0.20	99.80 ± 0.10	98.92 ± 1.67	99.99 ± 0.01	99.99 ± 0.01	**100.00 ± 0.00**	**100.00 ± 0.00**
3	96.81 ± 1.75	95.36 ± 2.28	88.51 ± 3.57	**100.00 ± 0.00**	**100.00 ± 0.00**	99.06 ± 1.32	99.99 ± 0.02	**100.00 ± 0.00**
4	98.72 ± 0.61	98.78 ± 0.30	95.06 ± 2.66	82.72 ± 3.62	93.70 ± 1.90	99.71 ± 0.26	95.92 ± 0.86	**99.81 ± 0.12**
5	95.96 ± 1.94	98.29 ± 0.47	94.15 ± 3.07	96.58 ± 1.31	94.91 ± 1.29	94.76 ± 2.25	95.98 ± 0.91	**97.45 ± 0.42**
6	99.50 ± 0.41	99.77 ± 0.14	98.83 ± 0.88	99.96 ± 0.08	**100.00 ± 0.00**	**100.00 ± 0.00**	**100.00 ± 0.00**	**100.00 ± 0.00**
7	99.44 ± 0.18	99.48 ± 0.11	98.34 ± 1.48	99.52 ± 0.45	99.77 ± 0.25	99.66 ± 0.24	**99.95 ± 0.04**	**99.95 ± 0.03**
8	89.97 ± 1.28	86.38 ± 3.12	81.03 ± 1.51	90.90 ± 1.58	87.79 ± 1.38	93.28 ± 4.67	98.50 ± 0.55	**99.63 ± 0.07**
9	99.04 ± 0.47	99.16 ± 0.26	98.84 ± 0.16	99.92 ± 0.13	99.82 ± 0.17	99.60 ± 0.45	99.86 ± 0.19	**100.00 ± 0.00**
10	85.27 ± 2.60	83.70 ± 1.11	81.16 ± 3.21	97.78 ± 2.31	99.61 ± 0.36	99.11 ± 0.64	99.40 ± 0.31	**99.87 ± 0.08**
11	90.35 ± 2.53	89.12 ± 2.30	82.84 ± 3.58	99.77 ± 0.29	**100.00 ± 0.00**	99.91 ± 0.13	99.94 ± 0.08	**100.00 ± 0.00**
12	99.55 ± 0.44	99.70 ± 0.10	98.41 ± 0.68	99.31 ± 0.53	**99.93 ± 0.07**	98.38 ± 1.33	99.83 ± 0.34	**99.93 ± 0.08**
13	96.98 ± 0.96	97.89 ± 1.68	95.45 ± 3.21	96.54 ± 2.19	99.93 ± 0.05	98.13 ± 0.09	99.49 ± 0.60	**99.96 ± 0.09**
14	92.94 ± 1.50	91.62 ± 1.94	93.15 ± 1.48	88.42 ± 4.46	95.84 ± 0.71	98.08 ± 0.94	96.75 ± 3.65	**99.75 ± 0.10**
15	47.86 ± 1.19	50.73 ± 4.21	52.43 ± 2.55	88.39 ± 4.00	98.13 ± 0.70	97.21 ± 1.81	98.04 ± 0.43	**99.80 ± 0.22**
16	94.80 ± 4.26	92.25 ± 2.29	88.56 ± 3.21	99.72 ± 0.24	99.83 ± 0.04	99.94 ± 0.08	99.55 ± 0.49	**100.00 ± 0.00**
OA (%)	88.78 ± 0.29	88.33 ± 0.40	86.25 ± 0.47	95.35 ± 0.35	96.63 ± 0.20	97.62 ± 0.73	98.97 ± 0.06	**99.73 ± 0.02**
AA (%)	92.75 ± 0.41	92.47 ± 0.23	90.23 ± 0.70	96.15 ± 0.20	98.06 ± 0.09	98.47 ± 0.18	98.95 ± 0.20	**99.72 ± 0.02**
Kappa × 100	87.45 ± 0.32	86.96 ± 0.44	84.64 ± 0.54	94.82 ± 0.39	96.26 ± 0.22	97.36 ± 0.80	98.85 ± 0.07	**99.70 ± 0.02**

**Table 8:** The categorized results of different methods on the Grass_dfc_2013 dataset.

Class	Methods							
	Conventional Classifiers			Classic Neural Networks				Proposed
	RBF-SVM	MLR	RF	2D-CNN	PyResNet	SSRN	HybridSN	
1	92.34 ± 5.15	86.39 ± 0.38	94.55 ± 2.19	92.19 ± 0.88	97.81 ± 0.69	98.29 ± 1.22	**98.35 ± 0.73**	98.06 ± 0.74
2	84.79 ± 7.71	94.47 ± 4.11	97.78 ± 0.85	95.67 ± 3.15	98.46 ± 0.50	97.59 ± 0.08	98.60 ± 0.74	**99.08 ± 0.23**
3	97.22 ± 1.30	99.70 ± 0.00	91.81 ± 2.02	93.67 ± 2.09	99.15 ± 0.57	98.69 ± 0.72	99.64 ± 0.20	**99.70 ± 0.06**
4	90.29 ± 2.11	97.11 ± 0.38	92.65 ± 1.39	92.64 ± 2.04	96.02 ± 1.16	97.77 ± 0.10	**99.60 ± 0.17**	97.8 ± 0.10
5	94.86 ± 1.68	99.13 ± 0.40	96.00 ± 2.36	99.97 ± 0.04	99.92 ± 0.12	99.83 ± 0.18	99.81 ± 0.27	**100.00 ± 0.00**
6	81.86 ± 1.30	82.20 ± 1.34	78.71 ± 3.77	72.05 ± 9.31	91.05 ± 2.57	87.92 ± 0.61	**96.96 ± 2.10**	94.5 ± 1.90
7	79.14 ± 4.57	85.61 ± 2.66	81.26 ± 4.36	75.00 ± 1.11	85.39 ± 2.60	91.43 ± 0.61	91.70 ± 1.40	**93.44 ± 0.25**
8	58.72 ± 7.63	50.73 ± 2.72	71.57 ± 2.09	57.82 ± 3.61	92.78 ± 0.52	90.24 ± 0.33	90.08 ± 0.70	**91.71 ± 0.79**
9	75.64 ± 6.74	70.39 ± 5.03	74.33 ± 2.81	58.30 ± 1.33	92.60 ± 0.74	97.20 ± 1.00	96.22 ± 1.65	**99.41 ± 0.61**
10	54.95 ± 11.71	52.92 ± 9.58	74.25 ± 4.64	69.33 ± 6.48	99.26 ± 0.54	**100.00 ± 0.00**	**99.91 ± 0.13**	**100.00 ± 0.00**
11	62.75 ± 6.41	56.71 ± 1.61	72.87 ± 1.81	79.91 ± 8.67	97.02 ± 0.61	99.46 ± 0.76	99.74 ± 0.26	**99.91 ± 0.07**
12	53.50 ± 10.72	47.12 ± 3.31	70.78 ± 4.91	86.43 ± 2.02	97.10 ± 0.78	**99.66 ± 0.00**	99.11 ± 0.48	99.57 ± 0.08
13	20.13 ± 7.94	4.03 ± 2.95	7.13 ± 2.48	9.34 ± 5.32	81.99 ± 4.99	89.46 ± 1.50	**94.93 ± 0.48**	94.84 ± 0.25
14	81.08 ± 12.99	82.60 ± 8.35	93.32 ± 3.23	92.77 ± 2.82	**100.00 ± 0.00**	**100.00 ± 0.00**	**100.00 ± 0.00**	**100 ± 0.00**
15	98.53 ± 0.44	98.81 ± 0.13	89.51 ± 3.56	**100.00 ± 0.00**	99.95 ± 0.08	99.84 ± 0.22	**100.00 ± 0.00**	**100 ± 0.00**
OA (%)	75.49 ± 0.71	74.57 ± 0.78	81.23 ± 0.60	80.10 ± 0.57	95.56 ± 0.51	96.96 ± 0.15	97.52 ± 0.30	**97.96 ± 0.13**
AA (%)	75.05 ± 0.90	73.80 ± 0.97	79.10 ± 0.84	78.34 ± 0.72	95.23 ± 0.64	96.49 ± 0.06	97.64 ± 0.32	**97.87 ± 0.18**
Kappa × 100	73.47 ± 0.77	72.45 ± 0.84	79.67 ± 0.66	78.45 ± 0.61	95.20 ± 0.55	96.72 ± 0.16	97.31 ± 0.32	**97.80 ± 0.14**

## Data Availability

Some or all data used during the study are available online in accordance with funder data retention polices. (http://www.ehu.eus/ccwintco/index.phptitle=Hyperspectral_Remote_Sensing_Scenes, https://hyperspectral.ee.uh.edu, accessed on 20 August 2021).
